# Association Between the Modified Renal Angina Index and Major Adverse Kidney Events at 90 Days in Critically Ill Adults: A Retrospective Cohort Study

**DOI:** 10.3390/diagnostics16203392

**Published:** 2026-10-19

**Authors:** Pınar Karabak Bilal, Hatice Şahin, Fatma Özkan Sipahioğlu, Gül Meral Kocabeyoğlu, Aşır Eren Seven, Mustafa Kemal Bayar

**Affiliations:** 1 Division of Intensive Care Medicine, Faculty of Medicine, Ankara University, Ankara 06100, Türkiye; mkbayar@yahoo.com; 2 Division of Nephrology, Ankara Etlik City Hospital, Ankara 06170, Türkiye; haticeharmansa@hotmail.com; 3 Department of Anesthesiology and Reanimation, Ankara Etlik City Hospital, Ankara 06170, Türkiye; ftmozkan_uzmdr@yahoo.com; 4 Division of Intensive Care Medicine, Ankara Etlik City Hospital, Ankara 06170, Türkiye; gmeralhosgoren@hotmail.com (G.M.K.); erenseven@hotmail.com (A.E.S.)

**Keywords:** acute kidney injury, major adverse kidney events, modified renal angina index, intensive care unit, renal risk stratification

## Abstract

**Background/Objectives:** Adult modified Renal Angina Index (mRAI) formulations predict acute kidney injury, but their association with major adverse kidney events at 90 days (MAKE90) remains unclear. We examined the association in critically ill adults without established chronic kidney disease (CKD). **Methods:** This retrospective cohort included 250 adults admitted to a tertiary intensive care unit (ICU) between December 2023 and April 2025. mRAI was calculated from data available at ICU admission. MAKE90 was defined as death, renal replacement therapy, or persistent renal dysfunction within 90 days. We used logistic regression, receiver operating characteristic analysis, and nested-model comparisons. **Results:** Admission mRAI was higher in patients with MAKE90 than in those without MAKE90 (median 10 [IQR 5–20] vs. 3 [IQR 1–6]; *p* < 0.001). After adjustment for SOFA score and sepsis, mRAI remained associated with MAKE90 (adjusted OR per point from the linear model, 1.073; 95% CI, 1.032–1.116). Spline analysis demonstrated a significant nonlinear association (*p* = 0.004), indicating that the per-point OR should be interpreted as a summary estimate rather than a constant effect across the score range. The AUC of mRAI was 0.752 (95% CI, 0.691–0.814), higher than that of admission AKI stage (0.653) and not significantly different from APACHE II (0.702) or SOFA (0.718). Adding mRAI to SOFA and sepsis raised the AUC from 0.735 to 0.782. **Conclusions:** In critically ill adults without established CKD, higher admission mRAI remained associated with MAKE90 after adjustment for SOFA score and sepsis and showed moderate discrimination. External validation in larger, independent cohorts is needed to confirm these findings.

## 1. Introduction

Acute kidney injury (AKI) is one of the most common complications in critically ill patients and is consistently associated with increased mortality, prolonged intensive care unit (ICU) and hospital stay, greater healthcare resource utilization, and an increased risk of chronic kidney disease (CKD) and long-term mortality. Importantly, the clinical consequences of AKI often extend well beyond the acute hospitalization, even in patients who experience apparent recovery of kidney function, highlighting the need for strategies that enable early identification of patients at increased risk of adverse renal outcomes. Consequently, early risk stratification has become a major focus of contemporary critical care nephrology [1,2,3].

Recognizing these limitations, recent AKI research has increasingly shifted from short-term changes in kidney function toward patient-centered outcomes that better reflect long-term prognosis [4]. Major adverse kidney events at 90 days (MAKE90), a composite endpoint consisting of all-cause mortality, renal replacement therapy (RRT), and persistent renal dysfunction, has emerged as a clinically meaningful outcome because it captures both survival and renal recovery after the acute phase of illness [5]. Early diagnosis of patients at high risk of MAKE90 can contribute to closer monitoring of these patients and the provision of more structured renal protective care [6].

Although serum creatinine remains the cornerstone of AKI diagnosis, it is an insensitive marker of early kidney injury because measurable changes often occur only after substantial loss of renal function. For this reason, increasing attention has been directed toward risk-stratification approaches that integrate baseline susceptibility with early changes in kidney function and clinical risk factors [7,8]. The Renal Angina Index (RAI), originally developed and validated in critically ill pediatric populations, combines patient risk factors with early changes in kidney function to identify individuals at increased risk of subsequent severe AKI [9,10].

Because the original RAI was designed specifically for children, several modified Renal Angina Index (mRAI) formulations have subsequently been proposed for adult ICU populations [5,11]. These adult formulations incorporate clinically relevant risk factors, including diabetes mellitus, vasopressor support, invasive mechanical ventilation, and early changes in serum creatinine, while preserving the conceptual framework of combining renal susceptibility with early kidney injury [5]. Previous studies have demonstrated that mRAI is associated with the development of moderate-to-severe AKI across heterogeneous adult ICU populations, including general intensive care, cardiac intensive care, and critically ill patients with coronavirus disease 2019 (COVID-19) [5,12,13]. Recent studies have shown that combining mRAI with urinary injury biomarkers, particularly urinary [TIMP-2] × [IGFBP7], may improve prediction of severe AKI, although validation in larger external cohorts is required [14].

However, the existing literature has primarily focused on the occurrence or severity of AKI during hospitalization [5,11,14]. To our knowledge, the association between admission mRAI and clinically meaningful longer-term kidney outcomes, particularly MAKE90, has not been adequately evaluated in a heterogeneous general adult ICU population. We hypothesized that the association between mRAI and renal outcomes might extend beyond the occurrence of AKI to longer-term outcomes such as MAKE90.

Therefore, the present study aimed to evaluate the association between admission mRAI and MAKE90 in critically ill adults, to assess its discriminative performance for identifying patients who subsequently develop MAKE90, and to determine whether this association persists after adjustment for conventional illness severity scores and clinically relevant covariates.

## 2. Materials and Methods

### 2.1. Study Design and Population

This retrospective observational cohort study included consecutive adult patients admitted to the tertiary intensive care unit of Etlik City Hospital between December 2023 and April 2025. For this study, ICU admission was defined as the time of the patient’s physical admission to the ICU rather than the time when the decision for ICU admission was made. The study was approved by the Ankara Etlik City Hospital Clinical Research Ethics Committee (Approval No. AEŞH-BADEK-2025-573; 3 December 2025) and was conducted in accordance with the principles of the Declaration of Helsinki. Given the retrospective design, the requirement for informed consent was waived.

Consecutive patients aged 18 years or older who had sufficient clinical and laboratory data for mRAI calculation and 90-day outcome assessment were included. Patients receiving chronic RRT before ICU admission were excluded. Patients with baseline estimated glomerular filtration rate (eGFR) < 60 mL/min/1.73 m^2^ and evidence of persistently reduced eGFR for at least 3 months were considered to have pre-existing CKD and were also excluded. This exclusion was intended to reduce the risk of misclassifying chronic kidney dysfunction as AKI; accordingly, the study population was limited to critically ill adults without established CKD.

Sepsis was defined according to Sepsis-3 criteria as suspected or documented infection associated with an acute increase in sequential organ failure assessment (SOFA) score of ≥2 points. Sepsis status was assessed independently of the primary reason for ICU admission; therefore, patients classified as having sepsis were not limited to those admitted to the ICU with sepsis as the primary diagnosis.

**Figure 1:**
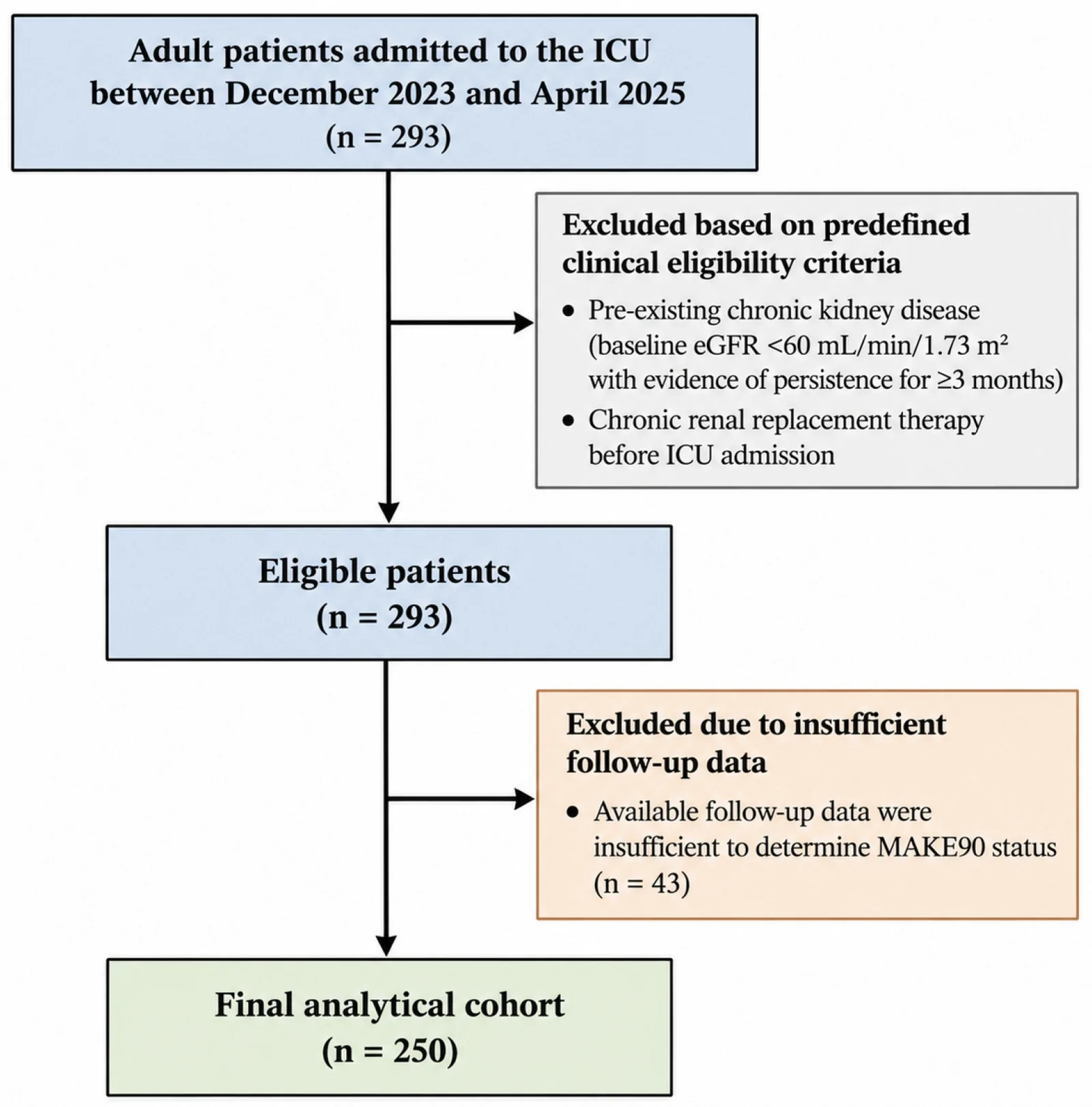
Study flow diagram. MAKE90 = Major Adverse Kidney Events at 90 days.

After application of the predefined clinical eligibility criteria, 293 patients were identified. Of these, 43 patients were excluded because 90-day follow-up data were unavailable after hospital discharge, preventing determination of MAKE90 status and leaving 250 patients in the final analytical cohort. The patient selection process is summarized in Figure 1.

### 2.2. Data Collection

Demographic characteristics, ICU admission diagnoses, laboratory findings, illness severity scores, requirements for RRT (acute hemodialysis or continuous renal replacement therapy), vasopressor support, and invasive mechanical ventilation, ICU length of stay, and 90-day clinical outcomes were collected retrospectively from the electronic medical record by review of routinely recorded clinical and laboratory information. Illness severity at ICU admission was assessed using the Acute Physiology and Chronic Health Evaluation II (APACHE II) and SOFA scores, which were routinely recorded at ICU admission.

Baseline laboratory variables included serum creatinine, blood urea, eGFR, albumin, lactate, C-reactive protein (CRP), and procalcitonin. eGFR was calculated by the hospital laboratory information system using the Chronic Kidney Disease Epidemiology Collaboration (CKD-EPI) equation [15].

### 2.3. Acute Kidney Injury Assessment

Acute kidney injury was diagnosed and staged according to the Kidney Disease: Improving Global Outcomes (KDIGO) serum creatinine criteria by comparing serum creatinine at hospital admission with the most recent stable pre-admission baseline value [16]. Because complete urine output data were not consistently available in this retrospective cohort, urine output criteria were not incorporated into AKI staging.

For AKI staging, baseline serum creatinine was defined as the most recent stable outpatient value obtained within 7–365 days before ICU admission. Patients were classified as having no AKI, stage 1 AKI, or stage 2 AKI according to the KDIGO serum creatinine criteria. No patients met KDIGO stage 3 AKI criteria at ICU admission.

### 2.4. Modified Renal Angina Index

The mRAI was calculated using an adult adaptation of the Renal Angina Index, based on the approach described by Matsuura et al. [17] and subsequent adult ICU studies [5,11]. The clinical condition score was assigned as follows: ICU admission without additional risk factors, 1 point; diabetes mellitus, 3 points; and vasopressor/inotropic support and/or invasive mechanical ventilation, 5 points. When more than one condition was present, the highest applicable score was used. The clinical condition component was determined exclusively from the patient’s status at ICU admission. Specifically, vasopressor/inotropic support and invasive mechanical ventilation were recorded according to whether these interventions were present at the time of ICU admission; interventions initiated later during the ICU stay were not incorporated into the mRAI.

The creatinine-based injury component was determined from the absolute change in serum creatinine between the pre-ICU measurement obtained in the emergency department or hospital ward and the first measurement obtained at ICU admission. For this purpose, the pre-ICU serum creatinine was defined as the last available creatinine measurement obtained before ICU admission, whereas the first ICU creatinine was the serum creatinine measured at ICU admission. This pre-ICU creatinine value was used specifically as the reference for mRAI calculation and was distinct from the historical baseline creatinine used for KDIGO AKI staging. Thus, these creatinine measurements served different methodological purposes: the historical stable outpatient creatinine represented baseline kidney function for AKI classification, whereas the most recent pre-ICU creatinine and the ICU admission creatinine captured the acute peri-admission change incorporated into the mRAI.

The injury score was assigned as follows: <0.10 mg/dL, 1 point; 0.10–0.29 mg/dL, 2 points; 0.30–0.39 mg/dL, 4 points; and ≥0.40 mg/dL, 8 points. The final mRAI score, ranging from 1 to 40, was calculated by multiplying the clinical condition and injury scores, with higher scores indicating greater renal risk.

**Figure 2:**
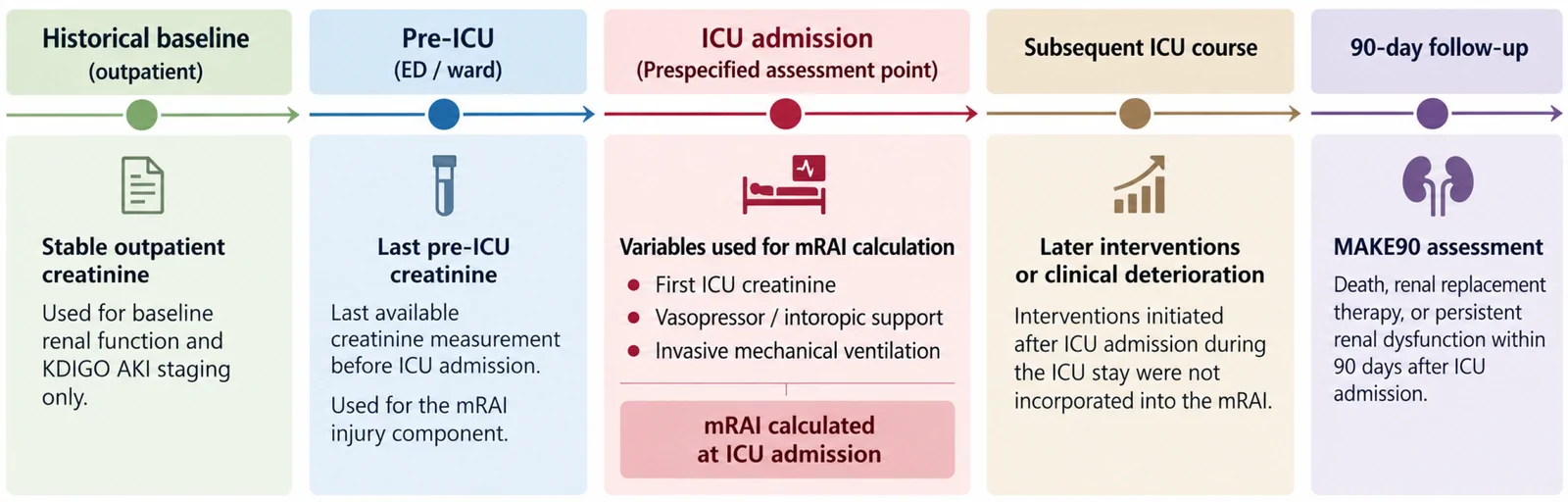
Temporal framework for mRAI assessment: The mRAI was calculated using variables available at ICU admission. Historical stable outpatient creatinine was used for baseline renal function and KDIGO AKI staging, whereas the last pre-ICU creatinine and the first creatinine measured at ICU admission were used to determine the mRAI injury component. Vasopressor/inotropic support and invasive mechanical ventilation reflected the patient’s status at ICU admission. Interventions initiated later during the ICU stay were not incorporated into the admission mRAI. MAKE90 was assessed during the 90-day follow-up period. AKI, acute kidney injury; ICU, intensive care unit; KDIGO, Kidney Disease: Improving Global Outcomes; MAKE90, major adverse kidney events within 90 days; mRAI, modified Renal Angina Index.

Accordingly, mRAI was calculated retrospectively using variables available at or before ICU admission, with no subsequent clinical deterioration or treatment escalation incorporated into the score. The temporal framework for mRAI assessment is illustrated in Figure 2.

### 2.5. Outcome Definition

The primary outcome was MAKE90, defined as the composite of all-cause mortality, RRT, or persistent renal dysfunction within 90 days after ICU admission. Patients meeting at least one of these components were classified as MAKE90-positive. Persistent renal dysfunction was defined as a >25% reduction in eGFR compared with the historical baseline renal function used for KDIGO AKI staging and was assessed among survivors using the serum creatinine measurement obtained closest to day 90 within a prespecified window of 90 ± 14 days.

Post-discharge mortality and RRT data were obtained from the hospital information management system and the national health record system (e-Nabız). Death occurring within 90 days and any RRT received during the 90-day follow-up period were considered MAKE90 events, regardless of whether RRT was temporary or ongoing at day 90. Patients discharged from the hospital without available 90-day follow-up data were considered to have indeterminate MAKE90 status and were excluded from the final analysis. MAKE90 components and their overlaps were summarized descriptively using mutually exclusive outcome categories.

### 2.6. Statistical Analysis

Continuous variables were assessed for distributional characteristics and are presented as median and interquartile range (IQR), as most variables showed non-normal distributions. Categorical variables are presented as numbers and percentages. Comparisons between patients with and without MAKE90 were performed using the Mann–Whitney U test for continuous variables and the chi-square test or Fisher’s exact test for categorical variables, as appropriate. Differences in admission mRAI scores across AKI stages were evaluated using the Kruskal–Wallis test and summarized using medians and interquartile ranges.

Because of the retrospective design, no prospective sample-size calculation was performed. The study sample was determined by all consecutive patients meeting the eligibility criteria during the predefined study period. The final cohort comprised 250 patients, including 108 MAKE90 events. The primary multivariable model was deliberately kept parsimonious and included three prespecified predictors to limit model complexity relative to the number of observed outcome events.

Binary logistic regression was used to evaluate variables associated with MAKE90. Univariable analyses included demographic characteristics, illness-severity scores, renal variables, and clinically relevant admission characteristics. The primary multivariable model included admission mRAI, SOFA score, and sepsis. SOFA and sepsis were selected a priori as clinically relevant covariates: SOFA was included to account for overall acute illness severity, whereas sepsis was included because of its association with AKI and adverse outcomes. Vasopressor requirement and invasive mechanical ventilation were not entered separately because they were components of the mRAI clinical-risk score. Results are reported as odds ratios (ORs) with 95% confidence intervals (CIs). To assess the robustness of the primary model, a sensitivity analysis was performed by additionally adjusting for age and admission eGFR.

Potential construct overlap between mRAI and conventional severity measures was examined using Spearman’s rank correlation coefficients between mRAI, SOFA, APACHE II, and admission AKI stage. Multicollinearity in the primary multivariable model was assessed using variance inflation factors (VIFs). Incremental predictive performance was evaluated by comparing a base model containing SOFA and sepsis with an extended model additionally containing mRAI. Differences in discrimination between these nested models were assessed using the DeLong test, model fit was compared using the likelihood-ratio test, and overall prediction error was assessed using the Brier score.

The functional form of the association between mRAI and MAKE90 was assessed by comparing the primary model assuming a linear mRAI effect with an otherwise identical model in which mRAI was modeled flexibly using a restricted cubic spline. The models were compared using a likelihood-ratio test. Because evidence of nonlinearity was identified, the per-point odds ratio from the linear model was retained as the coefficient from the prespecified primary model but was not interpreted as representing a constant effect across the entire mRAI range.

Model calibration was assessed using the calibration intercept and slope and graphically using a calibration plot with locally weighted smoothing (LOWESS). Internal validation of the multivariable model was performed using 1000 bootstrap resamples to estimate optimism-corrected model performance. The Brier score was used as an overall measure of prediction error.

The discriminative performance of mRAI, APACHE II, SOFA, and admission AKI stage for MAKE90 was evaluated using ROC curve analysis. AUCs and their 95% CIs were estimated using 1000 nonparametric bootstrap resamples. Exploratory cohort-derived thresholds were identified using the maximum Youden index, and sensitivity and specificity were calculated at the corresponding thresholds. The cohort-derived thresholds themselves were not subjected to internal validation and were not externally validated; therefore, they were not intended to define clinically actionable cutoffs. Pairwise comparisons of correlated AUCs were performed using DeLong’s method. All statistical tests were two-sided, and a *p* value < 0.05 was considered statistically significant. Statistical analyses were performed using Python version 3.12.

## 3. Results

### 3.1. Study Population

The study flow chart is illustrated in Figure 1. After application of the predefined clinical eligibility criteria, 293 patients were eligible for inclusion. Of these, 43 were subsequently excluded because available follow-up data were insufficient to determine MAKE90 status, leaving 250 patients in the final analytical cohort. Among the included patients, 108 (43.2%) developed MAKE90, whereas 142 (56.8%) did not experience the composite outcome.

The median age of the study population was 73.5 years (IQR, 61.2–82.0), and 115 patients (46.0%) were female. Median admission mRAI, APACHE II, and SOFA scores were 5 (IQR, 2–10), 17 (IQR, 12–24), and 5 (IQR, 3–7), respectively. At ICU admission, 148 patients (59.2%) had no AKI, 77 (30.8%) had stage 1 AKI, and 25 (10.0%) had stage 2 AKI. No patient met stage 3 AKI criteria at ICU admission. Overall baseline characteristics are presented in Table 1.

Clinical outcomes are summarized in Table 2. During ICU stay, 22 patients (8.8%) required RRT. The median ICU length of stay was 7 days (IQR, 5–13). ICU mortality occurred in 78 patients (31.2%). Overall, 108 patients (43.2%) fulfilled the MAKE90 definition.

The distribution of MAKE90 components is presented in Table 3. Among the 108 MAKE90-positive patients, 91 (84.3%) died, 26 (24.1%) required RRT, and 8 (7.4%) had persistent renal dysfunction. These components were not mutually exclusive.

### 3.2. Baseline Characteristics According to MAKE90 Status

Baseline demographic, clinical, and laboratory characteristics according to MAKE90 status are summarized in Table 4. Patients who developed MAKE90 had significantly higher APACHE II, SOFA, and mRAI scores (all *p* < 0.001). Sepsis, vasopressor requirement, invasive mechanical ventilation, and AKI at ICU admission were also more frequent among patients with MAKE90. Age and sex did not differ significantly between groups.

### 3.3. Association Between Admission mRAI and MAKE90

**Figure 3:**
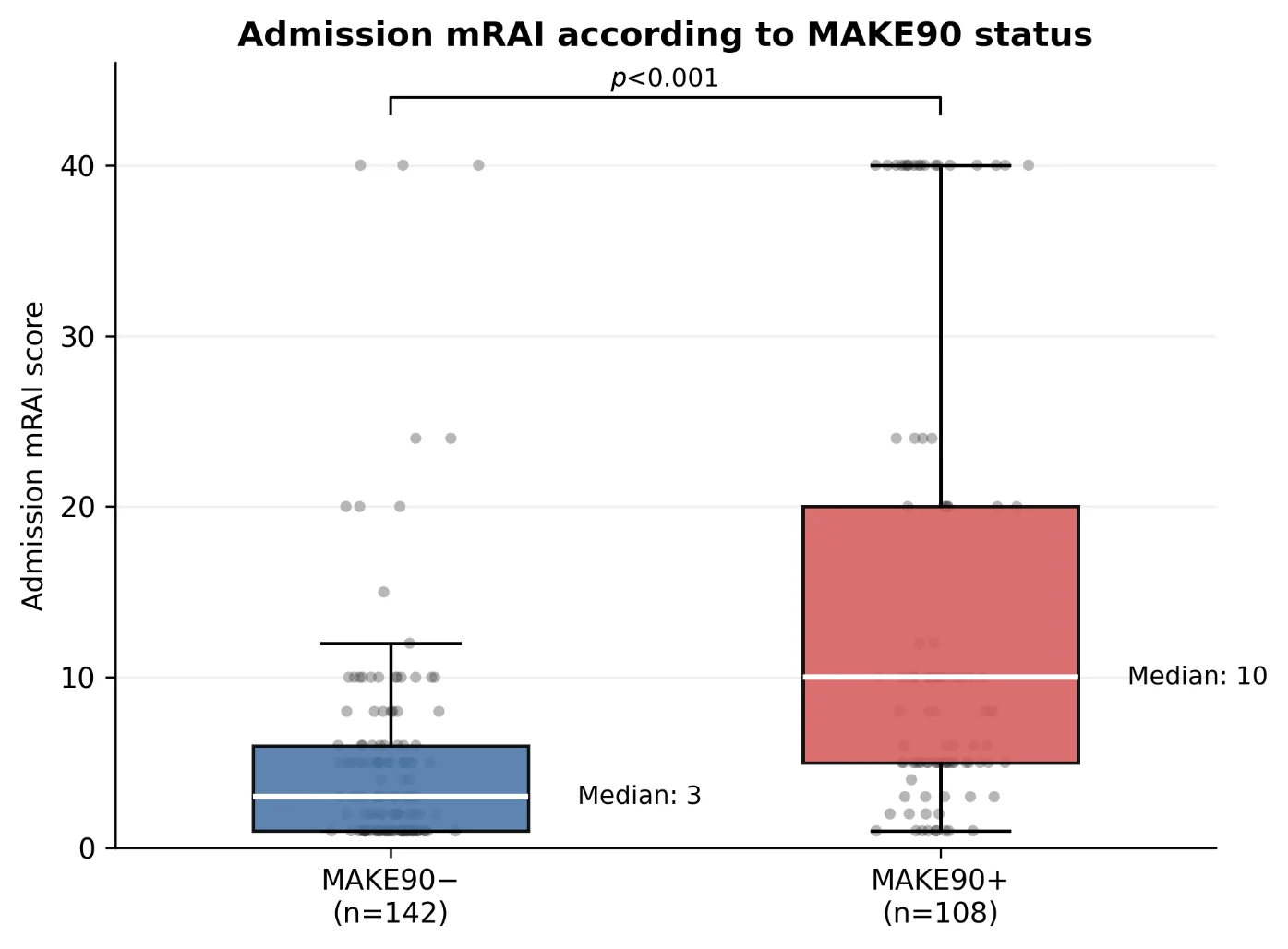
Distribution of admission modified Renal Angina Index scores according to MAKE90 status. Boxes represent the interquartile range, center lines indicate medians, whiskers extend to 1.5 times the interquartile range, and individual points represent patient-level observations.

Admission mRAI was significantly higher in patients who developed MAKE90 than in patients who remained free of the outcome (median, 10 [IQR, 5–20] vs. 3 [IQR, 1–6]; *p* < 0.001) (Figure 3).

The distribution of admission mRAI scores was as follows: 100 patients (40.0%) had scores of 1–4, 72 (28.8%) had scores of 5–9, 41 (16.4%) had scores of 10–19, and 37 (14.8%) had scores ≥ 20.

### 3.4. Association Between Admission mRAI and AKI Severity

**Figure 4:**
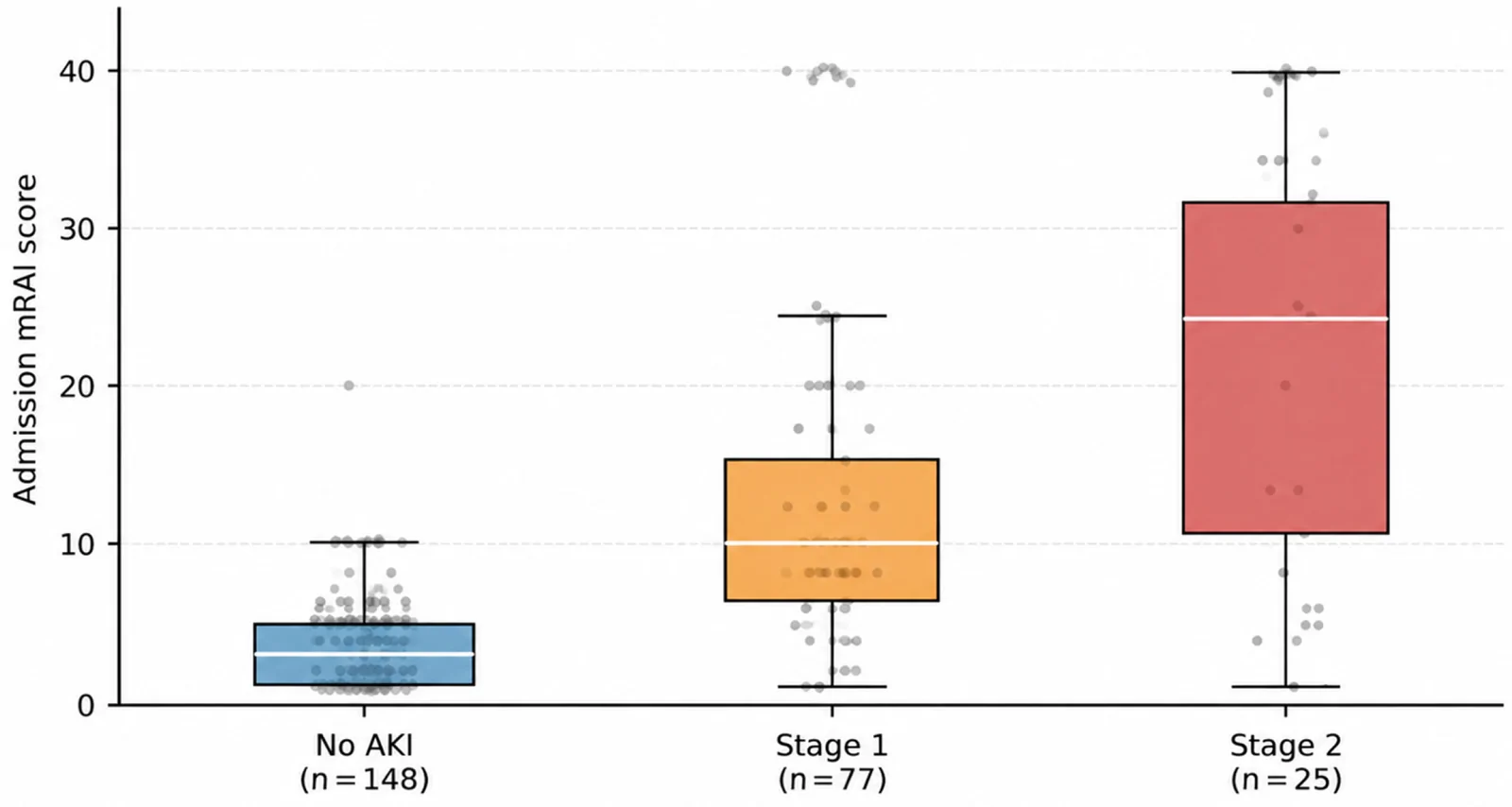
Distribution of admission modified Renal Angina Index scores across AKI stages. Box plots represent the median and interquartile range (IQR), with whiskers indicating values within 1.5 × IQR. Individual patient observations are shown as points. Admission mRAI scores differed significantly across AKI stages (Kruskal–Wallis test, H = 95.93; *p* < 0.001).

Admission mRAI increased progressively across AKI stages. Median mRAI was 3 (IQR, 1–5) in patients without AKI, 10 (IQR, 6–15) in patients with stage 1 AKI, and 24 (IQR, 8–40) in patients with stage 2 AKI (Kruskal–Wallis H = 95.93; *p* < 0.001) (Table 5 and Figure 4).

### 3.5. Subgroup Analysis in Patients Without AKI at ICU Admission

In a subgroup analysis restricted to the 148 patients without AKI at ICU admission, 45 (30.4%) developed MAKE90. Admission mRAI was higher in patients who developed MAKE90 than in those who did not (median, 5 [IQR, 3–6] vs. 2 [IQR, 1–5]; *p* < 0.001). Higher mRAI was also associated with MAKE90 in a univariable logistic regression model assuming a linear mRAI effect (OR per point, 1.255; 95% CI, 1.103–1.427; *p* < 0.001), with an AUC of 0.694 (95% CI, 0.599–0.783) (Table 6).

### 3.6. Logistic Regression Analyses and Incremental Model Performance for MAKE90

In univariable logistic regression analyses, higher age, mRAI, APACHE II, SOFA, admission AKI stage, serum creatinine, urea, and lactate were associated with increased odds of MAKE90. Sepsis, vasopressor requirement, and invasive mechanical ventilation were also associated with MAKE90, whereas higher albumin and eGFR were associated with lower odds of the outcome (Table 7).

In the primary multivariable model, admission mRAI remained associated with MAKE90 after adjustment for SOFA score and sepsis (adjusted OR, 1.073; 95% CI, 1.032–1.116; *p* < 0.001). This association remained significant in the sensitivity model additionally adjusted for age and admission eGFR (adjusted OR, 1.074; 95% CI, 1.028–1.123; *p* = 0.002) (Table 8). Given the statistically significant nonlinear association identified in the restricted cubic spline analysis, the per-point ORs reported in these linear logistic regression models should be interpreted as model-based summary estimates under the assumption of linearity on the log-odds scale, rather than as constant effects across the entire mRAI range.

Given the potential construct overlap between mRAI and conventional measures of illness severity and renal dysfunction, their relationships were further examined using correlation and multicollinearity analyses. mRAI was moderately correlated with SOFA (Spearman’s ρ = 0.392, *p* < 0.001), weakly correlated with APACHE II (ρ = 0.263, *p* < 0.001), and more strongly correlated with admission AKI stage (ρ = 0.617, *p* < 0.001). Variance inflation factors in the primary multivariable model were 1.21 for mRAI, 1.26 for SOFA, and 1.12 for sepsis, indicating no evidence of problematic multicollinearity. Although these findings indicate no substantial statistical multicollinearity, they do not exclude conceptual overlap between mRAI and SOFA.

Incremental predictive performance was evaluated by comparing a base model containing SOFA and sepsis with an extended model additionally containing mRAI. Among the 250 patients included in the analysis, the AUC increased from 0.735 for the base model to 0.782 for the extended model (ΔAUC = 0.047; 95% CI for the difference, 0.017–0.077; DeLong *p* = 0.002). Addition of mRAI also improved overall model fit (likelihood-ratio χ^2^ = 16.94, df = 1, *p* < 0.001) and reduced the Brier score from 0.202 to 0.188.

### 3.7. Discriminative Performance for MAKE90

**Figure 5:**
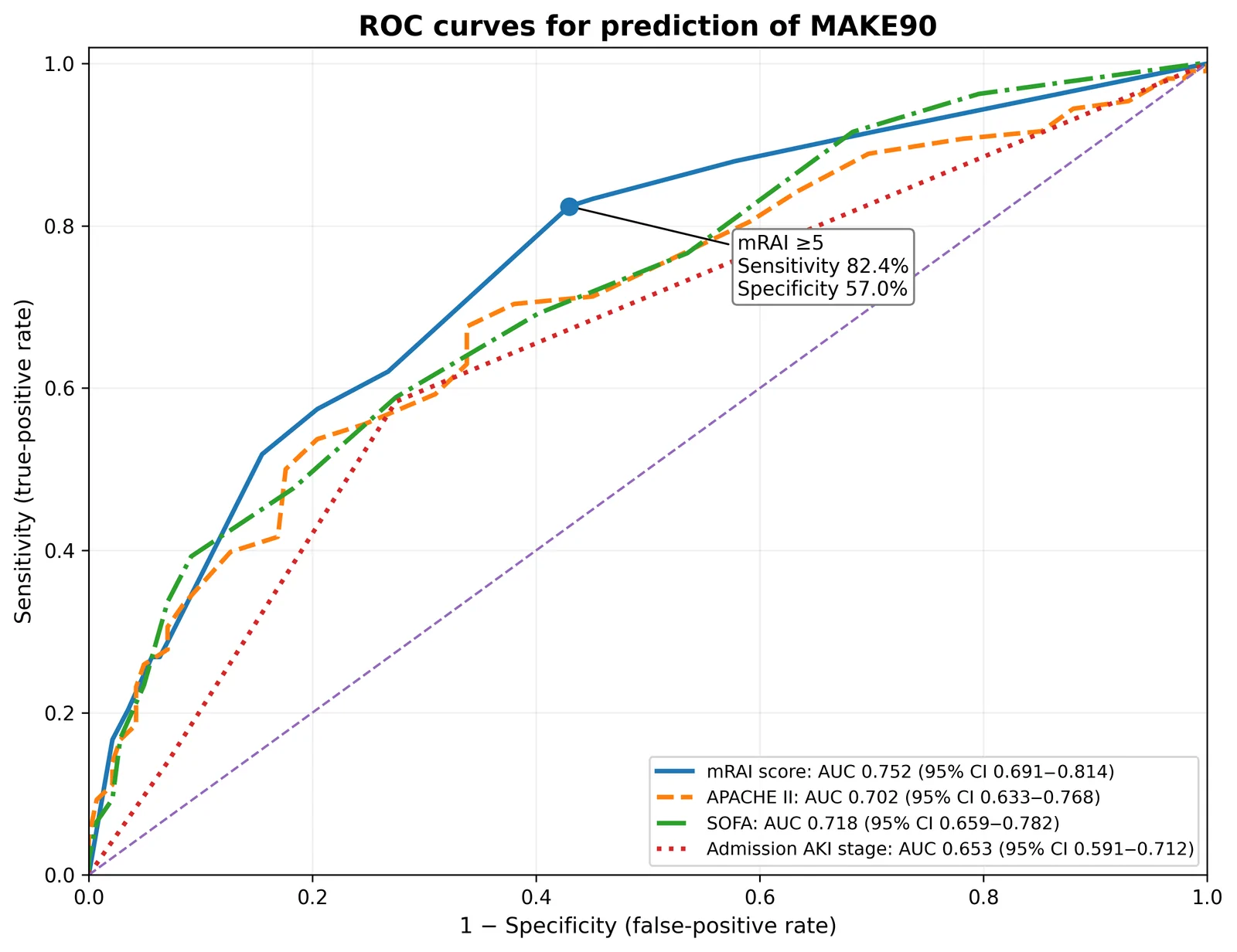
Receiver operating characteristic curves of admission mRAI, APACHE II, SOFA, and admission AKI stage for discrimination of MAKE90 status. Pairwise DeLong tests showed no significant difference between mRAI and APACHE II (*p* = 0.253) or SOFA (*p* = 0.359), whereas mRAI showed greater discrimination than admission AKI stage (*p* < 0.001). AUC, area under the receiver operating characteristic curve; MAKE90, major adverse kidney events at 90 days; mRAI, modified Renal Angina Index; SOFA, Sequential Organ Failure Assessment.

Admission mRAI demonstrated moderate discrimination for MAKE90, with an AUC of 0.752 (95% bootstrap CI, 0.691–0.814). An exploratory, cohort-derived threshold of mRAI ≥ 5 was identified using the maximum Youden index, with an apparent sensitivity of 82.4% and specificity of 57.0%. These estimates were derived and evaluated in the same cohort and should therefore not be interpreted as validated diagnostic performance. The discriminative performance of APACHE II and SOFA was comparable to that of mRAI, whereas admission AKI stage showed lower discrimination (Table 9 and Figure 5). Pairwise DeLong analysis confirmed that mRAI significantly outperformed admission AKI stage, whereas no significant differences were observed between mRAI and APACHE II or SOFA (Table 10).

The restricted cubic spline analysis demonstrated a statistically significant nonlinear association between admission mRAI and MAKE90. The spline-based model provided a significantly better fit than the corresponding model assuming a linear mRAI effect (likelihood-ratio χ^2^ = 11.16, *p* = 0.004). The spline model yielded an AUC of 0.800 and a Brier score of 0.179. These findings indicate that the association between mRAI and MAKE90 cannot be adequately characterized by a single constant per-point effect across the full mRAI range. Accordingly, the ORs obtained from the conventional linear logistic regression models should be regarded as summary estimates rather than uniform changes in the odds of MAKE90 at every mRAI level.

**Figure 6:**
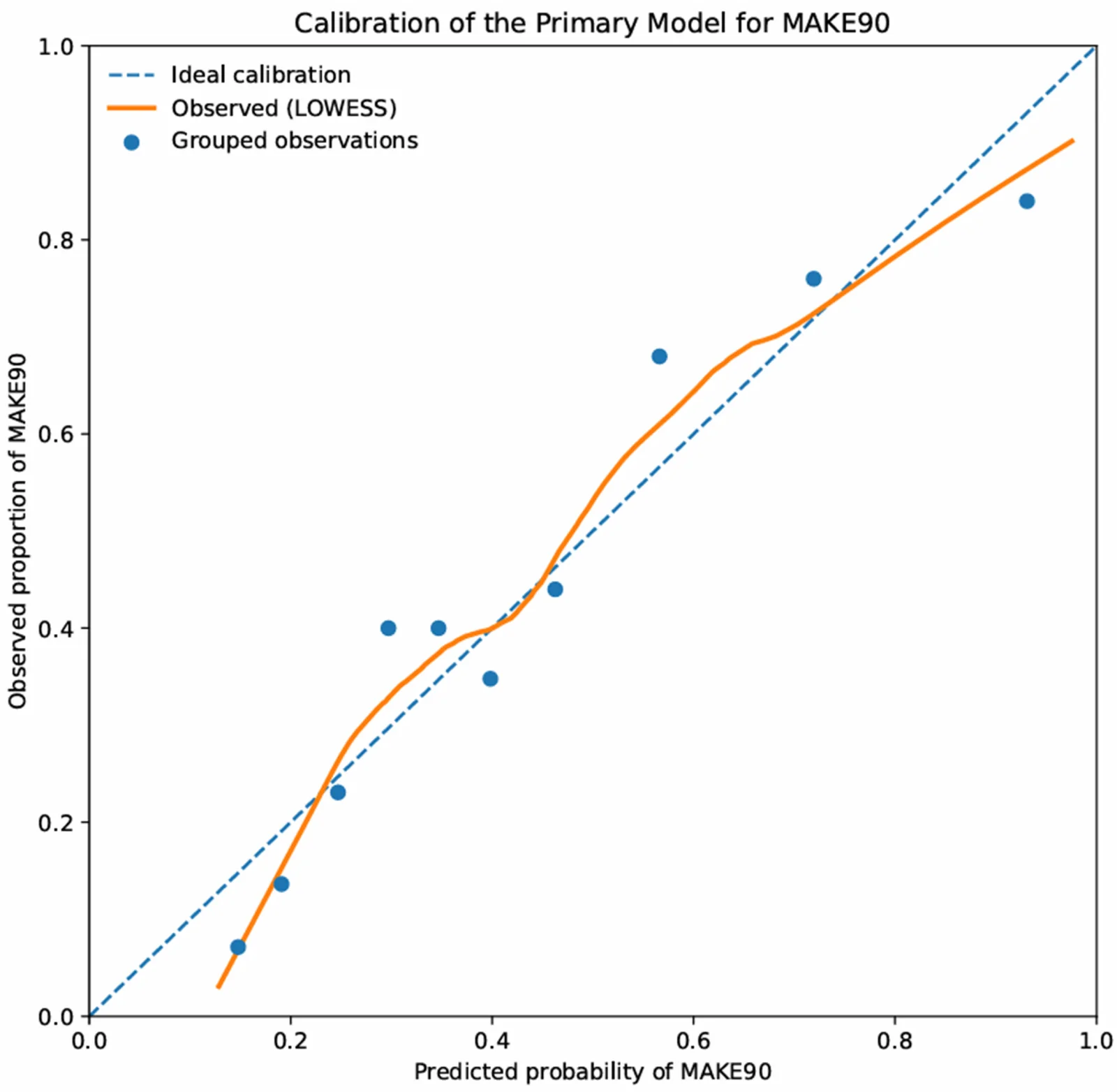
Calibration plot of the primary multivariable model for MAKE90. The primary model included admission mRAI, SOFA score, and sepsis. The dashed diagonal line represents perfect calibration, the solid line represents the apparent calibration relationship estimated using locally weighted smoothing (LOWESS), and points represent observed MAKE90 proportions across groups of predicted risk. The analysis included all 250 patients with complete data for all model variables. Internal validation was additionally performed using 1000 bootstrap resamples, as reported in the text.

Calibration of the primary multivariable model was also evaluated. Apparent calibration in the development cohort yielded an intercept of approximately 0 and a slope of 1.00. After internal validation using 1000 bootstrap resamples, the optimism-corrected calibration slope was 0.934, with an optimism-corrected AUC of 0.774 and Brier score of 0.195. The calibration plot is presented in Figure 6.

## 4. Discussion

In this retrospective cohort of critically ill adults, admission mRAI remained associated with MAKE90 after adjustment for SOFA score and sepsis and showed moderate discrimination for this composite outcome. However, direct pairwise comparisons showed no significant difference in discrimination between mRAI and either APACHE II or SOFA; therefore, these findings should not be interpreted as evidence that mRAI is superior to conventional severity scores. Rather, mRAI demonstrated comparable discrimination while incorporating kidney-related information alongside clinical features that partly overlap with conventional severity scores.

Our findings are consistent with previous studies demonstrating the value of renal angina–based models for predicting severe AKI in adult critically ill populations while extending their clinical relevance to kidney outcomes at 90 days. Across heterogeneous ICU settings, including general, cardiac, and COVID-19 intensive care populations, higher RAI or mRAI scores have consistently been associated with an increased risk of moderate-to-severe AKI. These findings have been reproduced in multiple adult cohorts using different adaptations of renal angina–based models, supporting the applicability of this risk-stratification approach across diverse critically ill populations [5,11,12,14,17].

The biological plausibility of the observed association between mRAI and MAKE90 is supported by current understanding of AKI pathophysiology. AKI involves inflammation, endothelial dysfunction, microvascular rarefaction, mitochondrial injury, oxidative stress, and maladaptive repair mechanisms that may persist beyond apparent biochemical recovery [1,2,18]. Contemporary mechanistic studies further implicate epigenetic reprogramming, G2/M cell-cycle arrest, cellular senescence, and persistent profibrotic signaling in maladaptive tubular repair, potentially promoting interstitial fibrosis, CKD progression, recurrent AKI, and adverse long-term outcomes [18,19].

The association between mRAI and admission AKI stage should be interpreted in the context of their shared creatinine-based components. Admission AKI stage reflects kidney dysfunction relative to the historical baseline creatinine, whereas mRAI combines the acute change between the pre-ICU and ICU admission creatinine measurements with clinical risk factors. Therefore, some overlap between the two measures is expected. Importantly, mRAI was also associated with MAKE90 among patients without AKI at ICU admission. This finding suggests that mRAI may identify differences in risk even when standard AKI criteria are not met at ICU admission. However, this subgroup analysis was exploratory and requires external validation. Similarly, Neyra et al. demonstrated the value of integrating multiple clinical variables for prediction of mortality and major adverse kidney events in critically ill patients with AKI [20].

MAKE90 includes mortality, renal replacement therapy, and persistent renal dysfunction and therefore captures both survival and renal recovery after critical illness. However, MAKE is not uniformly defined in the literature. In a 2024 review encompassing 122 studies, Maeda et al. showed significant heterogeneity in the definition of persistent renal dysfunction, dialysis-related components, and follow-up durations, with the most commonly used time points being 30 and 90 days [21]. In the present study, MAKE90 was defined as the occurrence of all-cause mortality, renal replacement therapy, or persistent renal dysfunction, with the latter defined as a >25% reduction in eGFR from baseline. Evidence that renal angina may be associated with outcomes beyond incident AKI is emerging. In a prospective cohort of 750 critically ill adults, Anas et al. reported an association between mRAI and MAKE30 [13]. In this study, admission mRAI remained associated with MAKE90 after adjustment for SOFA score and sepsis, extending the available evidence to a 90-day composite outcome. Unlike admission AKI stage, which characterizes kidney dysfunction at a specific time point, MAKE90 incorporates subsequent mortality, RRT, and persistent renal dysfunction, thereby providing a broader assessment of outcomes after critical illness [4,20,21].

Among the 108 MAKE90-positive patients, 91 (84.3%) died, indicating that the outcome was predominantly driven by mortality (Table 3). Because all-cause mortality is not kidney-specific, the association between mRAI and MAKE90 may partly reflect overall critical illness severity. The small number of renal events, particularly persistent renal dysfunction, limits conclusions about the kidney-specific prognostic contribution of mRAI.

Recent studies have also identified several readily obtainable clinical variables associated with medium-term kidney outcomes after critical illness. For example, fluid accumulation during the early ICU course has been associated with MAKE30 [22]. Biomarker-based approaches have also shown promising predictive performance but often require specialized assays that are not routinely available in many ICUs [14,23]. In contrast, mRAI is derived from routinely collected clinical variables [5,14].

APACHE II and SOFA primarily characterize overall illness severity and organ dysfunction [24,25], whereas mRAI integrates clinical susceptibility and acute illness characteristics with early changes in kidney function [5]. Nevertheless, mRAI incorporates vasopressor use and invasive mechanical ventilation, which conceptually overlap with the cardiovascular and respiratory components of SOFA. Although the VIF analysis showed no evidence of problematic multicollinearity, this does not eliminate the underlying conceptual overlap between the two scores. Adding mRAI to a base model containing SOFA and sepsis improved discrimination and model fit and reduced prediction error in this cohort. These findings suggest that mRAI provides additional prognostic information beyond SOFA and sepsis within the study cohort, rather than demonstrating an entirely independent kidney-specific effect. However, this improvement does not establish clinical utility, which requires external validation.

An important finding of this study was the nonlinear association between admission mRAI and MAKE90. The restricted cubic spline analysis demonstrated significantly better model fit than the conventional linear specification, suggesting that the association varies across the mRAI range. Consequently, the adjusted OR per one-point increase in mRAI should be interpreted as a summary estimate from the linear logistic regression model rather than as a constant effect applicable to all score values. This distinction is particularly relevant because mRAI is calculated by multiplying categorical clinical-risk and creatinine-based injury scores, resulting in a discrete and unevenly spaced scoring scale. Further studies are needed to determine whether this nonlinear relationship is reproducible in independent cohorts.

Internal bootstrap validation yielded an optimism-corrected calibration slope of 0.934, indicating some model optimism. These findings require confirmation in an independent cohort.

### Strengths and Limitations

The strengths of this study include the evaluation of the clinically meaningful MAKE90 endpoint and the comparison of mRAI with both established ICU severity scores and admission AKI stage. This study has several limitations.

First, the retrospective single-center design may limit generalizability and precludes causal inference. The retrospective design also prevented reliable assessment of dehydration status and contrast-associated AKI, both of which may have contributed to renal dysfunction. AKI ascertainment was based on serum creatinine criteria only because reliable urine output data were not available. In addition, AKI was assessed at presentation by comparing admission serum creatinine with the most recent stable pre-admission baseline value; therefore, AKI severity based on serial in-hospital creatinine trajectories could not be evaluated. Second, 43 otherwise eligible patients were excluded because available follow-up data were insufficient to determine MAKE90 status. Because loss to follow-up may not have occurred at random, selection bias cannot be excluded. The observed MAKE90 incidence and associated effect estimates may therefore not fully represent the entire eligible population. Third, patients with established CKD were excluded to reduce the risk of misclassifying chronic kidney dysfunction as AKI. This limits the generalizability of our findings, which should therefore be considered primarily applicable to critically ill adults without established CKD.

Fourth, MAKE90 includes all-cause mortality and is therefore not an exclusively kidney-specific endpoint; it may partly reflect overall critical illness severity. Although the frequencies and overlaps of MAKE90 components were identified, component-specific multivariable analyses were not performed because of the limited number of renal events, particularly persistent renal dysfunction. Therefore, the extent to which the observed association between mRAI and MAKE90 reflects kidney-specific outcomes rather than overall illness severity remains uncertain. Fifth, the mRAI threshold of ≥5 was derived and evaluated in the same cohort using the maximum Youden index. The threshold should therefore be considered exploratory. Its sensitivity and specificity are apparent estimates and may be optimistic and unstable in this relatively small cohort. The threshold itself was not internally or externally validated and should not be interpreted as a clinically actionable cutoff.

Finally, although bootstrap internal validation was performed for the multivariable model, model development and evaluation were conducted within the same single-center cohort. The reported discrimination, calibration, and incremental performance may therefore still be optimistic. External validation is required before clinical implementation.

## 5. Conclusions

In this retrospective cohort of critically ill adults without established CKD, higher admission mRAI scores remained associated with MAKE90 after adjustment for SOFA score and sepsis and demonstrated moderate discrimination for this composite outcome. mRAI showed greater discrimination than admission AKI stage, while its discrimination did not differ significantly from APACHE II or SOFA. Adding mRAI to a model containing SOFA and sepsis improved model performance within this cohort. However, these findings represent internal evidence and do not establish clinical utility. External validation in independent cohorts is required before mRAI can be considered for routine clinical risk stratification.

## Figures and Tables

**Table 1:** Baseline demographic and clinical characteristics of the study cohort.

Characteristic	Overall Cohort (*n* = 250)
**Demographics**	
Age, years	73.5 (61.2–82.0)
Female sex, *n* (%)	115 (46.0)
**ICU admission diagnosis, *n* (%)**	
Pulmonary	126 (50.4)
Sepsis	35 (14.0)
Neurological	30 (12.0)
Cardiovascular	15 (6.0)
Abdominal	15 (6.0)
Metabolic	6 (2.4)
Drug intoxication	6 (2.4)
Trauma	5 (2.0)
Other	12 (4.8)
**Illness severity**	
mRAI score	5 (2–10)
APACHE II score	17 (12–24)
SOFA score	5 (3–7)
**Renal status at ICU admission**	
No AKI, *n* (%)	148 (59.2)
AKI stage 1, *n* (%)	77 (30.8)
AKI stage 2, *n* (%)	25 (10.0)
**Clinical characteristics**	
Sepsis, *n* (%)	120 (48.0)
Vasopressor requirement, *n* (%)	81 (32.4)
Invasive mechanical ventilation requirement, *n* (%)	95 (38.0)

Continuous variables are presented as median (IQR), and categorical variables as *n* (%). Admission diagnosis refers to the primary reason for ICU admission, whereas sepsis under clinical characteristics indicates the presence of sepsis at ICU admission irrespective of the primary admission diagnosis. AKI, acute kidney injury; APACHE II, Acute Physiology and Chronic Health Evaluation II; ICU, intensive care unit; IQR, interquartile range; mRAI, modified Renal Angina Index; SOFA, Sequential Organ Failure Assessment.

**Table 2:** Clinical outcomes during ICU stay and at 90-day follow-up.

Outcome	*n* (%) or Median (IQR)
ICU length of stay, days median (IQR)	7 (5–13)
Renal Replacement Therapy, *n* (%)	22 (8.8)
MAKE90, *n* (%)	108 (43.2)
ICU mortality, *n* (%)	78 (31.2)

ICU, intensive care unit; IQR, interquartile range; MAKE90, major adverse kidney events at 90 days; renal replacement therapy included intermittent hemodialysis and/or continuous renal replacement therapy.

**Table 3:** Distribution of MAKE90 components during the 90-day follow-up period.

MAKE90 Outcome Category	*n* (%)
Death without RRT	76 (70.4)
RRT requirement and death	15 (13.9)
RRT without death or persistent renal dysfunction	9 (8.3)
Persistent renal dysfunction, with or without prior RRT	8 (7.4)
**Total MAKE90-positive patients**	**108 (100.0)**

Categories are mutually exclusive. Two patients with persistent renal dysfunction also received RRT. Overall, 91 patients died, 26 received RRT, and 8 had persistent renal dysfunction. Percentages are based on 108 MAKE90-positive patients. RRT, renal replacement therapy.

**Table 4:** Baseline demographic, clinical, and laboratory characteristics according to MAKE90 status.

Variable	Overall (*n* = 250)	MAKE90-Negative (*n* = 142)	MAKE90-Positive (*n* = 108)	*p* Value
**Baseline characteristics**				
Age, years	73.5 (61.2–82.0)	72.0 (60.0–81.0)	76.0 (65.0–82.2)	0.076
Female sex	115 (46.0)	67 (47.2)	48 (44.4)	0.667
APACHE II	17.0 (12.0–24.0)	15.0 (11.0–20.0)	22.5 (15.0–29.0)	<0.001
SOFA score	5.0 (3.0–7.0)	4.0 (2.0–6.0)	6.0 (4.0–9.0)	<0.001
mRAI score	5.0 (2.0–10.0)	3.0 (1.0–6.0)	10.0 (5.0–20.0)	<0.001
**Laboratory variables**				
Serum creatinine at ICU admission, mg/dL	0.9 (0.7–1.3)	0.9 (0.6–1.1)	1.1 (0.7–1.5)	<0.001
Serum urea, mg/dL	59.3 (35.5–82.6)	48.3 (32.4–71.2)	69.6 (44.3–100.2)	<0.001
eGFR, mL/min/1.73 m^2^	73.0 (47.2–96.8)	78.5 (58.0–102.5)	57.0 (40.0–88.5)	<0.001
Albumin, g/dL	3.1 (2.7–3.6)	3.3 (2.8–3.7)	3.0 (2.6–3.4)	<0.001
Procalcitonin, ng/mL	0.4 (0.1–1.6)	0.2 (0.1–1.1)	0.8 (0.2–2.8)	<0.001
CRP, mg/L	77.8 (26.3–163.0)	56.9 (12.4–155.8)	104.0 (47.5–169.5)	0.004
Lactate, mmol/L	2.1 (1.6–2.9)	1.9 (1.6–2.6)	2.5 (1.8–3.4)	<0.001
**Clinical characteristics**				
Sepsis	120 (48.0)	52 (36.6)	68 (63.0)	<0.001
Vasopressor requirement	81 (32.4)	20 (14.1)	61 (56.5)	<0.001
Invasive mechanical ventilation	95 (38.0)	27 (19.0)	68 (63.0)	<0.001
**AKI stage at ICU admission ** **<0.001**				
No AKI at admission	148 (59.2)	103 (72.5)	45 (41.7)	
AKI stage 1 at admission	77 (30.8)	29 (20.4)	48 (44.4)	
AKI stage 2 at admission	25 (10.0)	10 (7.0)	15 (13.9)	

Continuous variables are shown as median (IQR); categorical variables as *n* (%). AKI: acute kidney injury; CRP: C-reactive protein; eGFR: estimated glomerular filtration rate; IQR: interquartile range; MAKE90: major adverse kidney events at 90 days; mRAI: modified Renal Angina Index.

**Table 5:** Distribution of admission mRAI scores across AKI stages.

Admission AKI Stage	*n*	mRAI, Median (IQR)	Range
No AKI	148	3 (1–5)	1–20
AKI stage 1	77	10 (6–15)	1–40
AKI stage 2	25	24 (8–40)	1–40

Kruskal–Wallis H = 95.93; *p* < 0.001. AKI, acute kidney injury; IQR, interquartile range; mRAI, modified Renal Angina Index.

**Table 6:** Prognostic performance of admission mRAI among patients without AKI at ICU admission.

Parameter	Result
Patients, n/MAKE90, *n* (%)	148/45 (30.4)
mRAI, MAKE90-positive vs. MAKE90-negative, median (IQR)	5 (3–6) vs. 2 (1–5); *p* < 0.001
OR per 1-point increase in mRAI (95% CI)	1.255 (1.103–1.427); *p* < 0.001
AUC (95% CI)	0.694 (0.599–0.783)

AUC, area under the receiver operating characteristic curve; CI, confidence interval; IQR, interquartile range; MAKE90, major adverse kidney events at 90 days; mRAI, modified Renal Angina Index; OR, odds ratio.

**Table 7:** Univariable logistic regression analyses of factors associated with MAKE90.

Variable	OR	95% CI	*p* Value
Age, per 1-year increase	1.018	1.002–1.036	0.032
mRAI score, per 1-point increase	1.097	1.057–1.139	<0.001
APACHE II, per 1-point increase	1.096	1.059–1.133	<0.001
SOFA, per 1-point increase	1.307	1.192–1.434	<0.001
Sepsis, present vs. absent	2.942	1.751–4.943	<0.001
Vasopressor requirement, present vs. absent	7.917	4.315–14.525	<0.001
Invasive mechanical ventilation, present vs. absent	7.241	4.083–12.841	<0.001
Admission AKI stage, per 1-stage increase	2.385	1.597–3.561	<0.001
Admission creatinine, per 1 mg/dL increase	2.775	1.589–4.847	<0.001
Urea, per 10 mg/dL increase	1.149	1.072–1.243	<0.001
Albumin, per 1 g/dL increase	0.470	0.299–0.739	0.001
eGFR, per 10 mL/min/1.73 m^2^ increase	0.869	0.793–0.942	<0.001
Lactate, per 1 mmol/L increase	1.443	1.173–1.775	<0.001

For continuous variables, ORs are reported for the increment specified in the variable label. For binary variables, ORs represent presence versus absence. CI, confidence interval; eGFR, estimated glomerular filtration rate; MAKE90, major adverse kidney events at 90 days; mRAI, modified Renal Angina Index; OR, odds ratio; SOFA, Sequential Organ Failure Assessment.

**Table 8:** Multivariable logistic regression models for MAKE90.

Variable	Primary Model, Adjusted OR (95% CI)	*p* Value	Sensitivity Model, Adjusted OR (95% CI)	*p* Value
mRAI score	1.073 (1.032–1.116)	<0.001	1.074 (1.028–1.123)	0.002
SOFA score	1.200 (1.086–1.326)	<0.001	1.197 (1.082–1.325)	<0.001
Sepsis	1.874 (1.049–3.346)	0.034	1.943 (1.071–3.526)	0.029
Age, per 1-year increase	—	—	1.019 (0.997–1.042)	0.096
Admission eGFR, per 1 mL/min/1.73 m^2^ increase	—	—	1.002 (0.990–1.014)	0.779

The primary model included mRAI, SOFA, and sepsis; the sensitivity model additionally included age and admission eGFR. The per-point mRAI ORs are summary estimates from models assuming linearity, given the observed nonlinearity. CI, confidence interval; eGFR, estimated glomerular filtration rate; MAKE90, major adverse kidney events at 90 days; mRAI, modified Renal Angina Index; OR, odds ratio; SOFA, Sequential Organ Failure Assessment.

**Table 9:** Discriminative performance of admission scores and AKI stage for MAKE90.

Predictor	AUC	95% CI	Threshold	Sensitivity	Specificity	Youden
mRAI score	0.752	0.691–0.814	≥5	82.4%	57.0%	0.394
APACHE II	0.702	0.633–0.768	≥18	67.6%	66.2%	0.338
SOFA	0.718	0.659–0.782	≥6	58.9%	72.5%	0.314
Admission AKI stage	0.653	0.591–0.712	≥1	58.3%	72.5%	0.309

AUC confidence intervals were estimated using 1000 nonparametric bootstrap resamples. Thresholds were selected using the maximum Youden index within the same cohort and should be considered exploratory. The reported sensitivity and specificity are apparent estimates and have not been internally or externally validated. AKI, acute kidney injury; AUC, area under the ROC curve; CI, confidence interval; mRAI, modified Renal Angina Index; ROC, receiver operating characteristic.

**Table 10:** Pairwise comparisons of correlated AUCs for MAKE90 using the DeLong method.

Comparison	AUC Difference	*p* Value
mRAI score vs. APACHE II	0.050	0.253
mRAI score vs. SOFA	0.035	0.359
APACHE II vs. SOFA	−0.018	0.581
mRAI score vs. Admission AKI stage	0.100	<0.001

AUC differences were calculated from paired ROC curves. AUC, area under the ROC curve.

## Data Availability

The data presented in this study are available on request from the corresponding author.

## Abbreviations

The following abbreviations are used in this manuscript:

AKI	Acute Kidney Injury
APACHE II	Acute Physiology and Chronic Health Evaluation II
AUC	Area Under the Curve
CI	Confidence Interval
CKD	Chronic Kidney Disease
CKD-EPI	Chronic Kidney Disease Epidemiology Collaboration
CRP	C-Reactive Protein
eGFR	Estimated Glomerular Filtration Rate
ICU	Intensive Care Unit
IQR	Interquartile Range
KDIGO	Kidney Disease: Improving Global Outcomes
MAKE90	Major Adverse Kidney Events at 90 Days
mRAI	Modified Renal Angina Index
OR	Odds Ratio
RAI	Renal Angina Index
ROC	Receiver Operating Characteristic
RRT	Renal Replacement Therapy
SOFA	Sequential Organ Failure Assessment
